# Semen parameters after SARS‐CoV‐2 infection: A literature review

**DOI:** 10.1002/hsr2.745

**Published:** 2022-08-10

**Authors:** Kathrine Tufvesson, Laura Catalini, Jens Fedder

**Affiliations:** ^1^ Research Unit of Reproductive Medicine, Faculty of Health Sciences University of Southern Denmark Odense Denmark; ^2^ Centre of Andrology and Fertility Clinic Dept. D, Odense University Hospital Odense Denmark

**Keywords:** COVID‐19, male infertility, SARS‐CoV‐2, semen parameters, spermatogenesis

## Abstract

**Background and Aims:**

The severe acute respiratory syndrome coronavirus 2 (SARS‐CoV‐2) is known to affect multiple organs by binding to angiotensin‐converting enzyme 2 receptors and might therefore affect male fertility. This review aims to collect all original articles on the effects of SARS‐CoV‐2 infection on male fertility, including the duration of time after infection required for these effects to begin to manifest and recommend how clinicians should approach cases with a recent illness.

**Methods:**

This review was developed according to the preferred reporting items for systematic reviews and meta‐analyses guidelines. The search string was applied to four online databases—namely Pubmed, Embase, Medline, and the Cochrane COVID‐19 Register—and screened using the online tool Covidence.org. Articles were eligible for inclusion if they were cohort studies involving a healthy male population diagnosed with COVID‐19, each of whom had semen samples collected before and after the infection or two different semen samples collected after the diagnosis.

**Results:**

Nine cohort studies were eventually included. Five articles had pre‐ and post‐COVID‐19 data while four had two sets of post‐COVID‐19 data. The three largest studies found a statistically significant decrease in all semen parameters when waiting less than 3 months from diagnosis before sample collection, and no significant differences in results when the ejaculate was analyzed more than 3 months after recovery. One study compared the COVID‐19 patients with a control group and found a significant decrease in semen parameters in the COVID‐19 group.

**Conclusion:**

Spermatogenesis seems to be affected by SARS‐CoV‐2 infection, but the impact tends to reverse within 3–4 months. It is still unclear why male fertility is affected by SARS‐CoV‐2 infection, and it might be the result of several different components. Clinicians should consider recent SARS‐CoV‐2 infection as a possible reason for the low semen quality of patients' semen samples, and might therefore need to collect new samples after 4 months before further treatment.

## 1 INTRODUCTION

Over 2 years have passed since the severe acute respiratory syndrome coronavirus 2 (SARS‐CoV‐2) was first discovered.^1^ In just a few months, it spread to the entire world, resulting in the death of around 5½ million people, and to date, almost 3 billion cases of coronavirus 2019 (COVID‐19) have been reported.^2^ As a result of the pandemic caused by the virus, researchers all over the whole started exploring various ways the disease affected people and could be transmitted. It was discovered that people reacted very differently to infection with the virus—some people got critically ill and others were just asymptomatic carriers.^3^

Parallel with research efforts, some countries shut down noncritical hospital wards, including fertility clinics,^4^ to limit the spread of the virus.^5^ At the same time, the European Society of Human Reproduction and Embryology started collecting relevant research and knowledge of the area to provide guidelines for fertility clinics.^6^

It became clear at the outbreak of the virus that it affects more than just the respiratory system.^3^ Being similar to viruses that caused past pandemics such as the SARS and the Middle East respiratory syndrome, researchers already had hypotheses about how this novel SARS‐CoV‐2 could affect reproduction.^3^

Acute illnesses with febrilia might affect spermatogenesis for a limited time,^7^ which means that since SARS‐CoV‐2 infection often presents with this symptom,^3^ this effect might also occur in this case.

Another theory builds on the fact that the SARS‐CoV‐2 viral genome enters the host cell via spike proteins, binding to the angiotensin‐converting enzyme 2 (ACE2) receptor which is located in different cells in the body, including the lungs just like the former coronaviruses.^4^ ACE2 is also expressed selectively in Leydig cells in the male genitals, suggesting that it can affect male reproduction.^4^ Even if reversible, spermatogenesis takes about 74 days, giving a window of about 3 months within which a man could briefly fulfill the criteria for assisted reproductive treatment (ART) by falling under the reference values established by the World Health Organization (WHO).^8^

Earlier reviews mostly address if COVID‐19 affects male fertility or can be found in semen.

To our knowledge, no review has yet collected studies that compare changes in an individual's fertility status in connection to COVID‐19.

The aim of this review is to evaluate how SARS‐CoV‐2 infection affects semen quality and male fertility. Further, this review aims to assess how long the semen quality might be affected if a connection is found between the virus and semen quality.

Clinicians planning ART for patients who are or have been SARS‐CoV‐2‐positive will find this knowledge relevant.

## 2 MATERIALS AND METHODS

### 2.1 Search string

COVID‐19 is a relatively new disease, and the amount of research on the subject is limited. Four different electronic databases were searched. An advanced search was first made on pubmed.org, resulting in the following search string comprising of keywords from the main aim of this review: *((((corona virus 2019) OR (sars‐cov‐2)) OR (covid19)) AND ((((semen) OR (sperm)) OR (spermatozoa)) OR (spermatid)))*. Afterward, the same words were used to conduct an expanded search on Embase and Medline including both keywords and terms. Lastly, the search string from PubMed was used in the Cochrane COVID‐19 register. All four searches were made on the same day in September 2021.

### 2.2 Criteria

To get an idea of how COVID‐19 affects semen quality, we focused on publications about men with data on their semen quality before infection. Therefore, all included articles were cohort studies involving male populations diagnosed with COVID‐19, each of whom underwent preinfection and postinfection semen quality analyses. Because of the limited number of studies on COVID‐19 and male fertility, the search string could not be too specific or we would have missed some articles.

Only a few articles were found during the full‐text screening, as a result of which the inclusion criteria above were expanded. Studies with no data before COVID‐19 infection but with more than one analyzed semen sample from each patient after the diagnosis of COVID‐19 were included. This made it possible to observe the potential changes in semen quality over time after infection.

### 2.3 Screening

The results from the searches were screened using the online tool Covidence.org, which removed duplicates automatically. Title and abstract screening were conducted by one investigator twice, with an interval of a couple of days in between, without knowing the results from the first time to make sure that no article was overlooked. The full‐text screening was conducted by one investigator, with support from another investigator if the relevance of an article to the review was unclear. The final articles were all read by two investigators.

### 2.4 Search results

**Figure 1:**
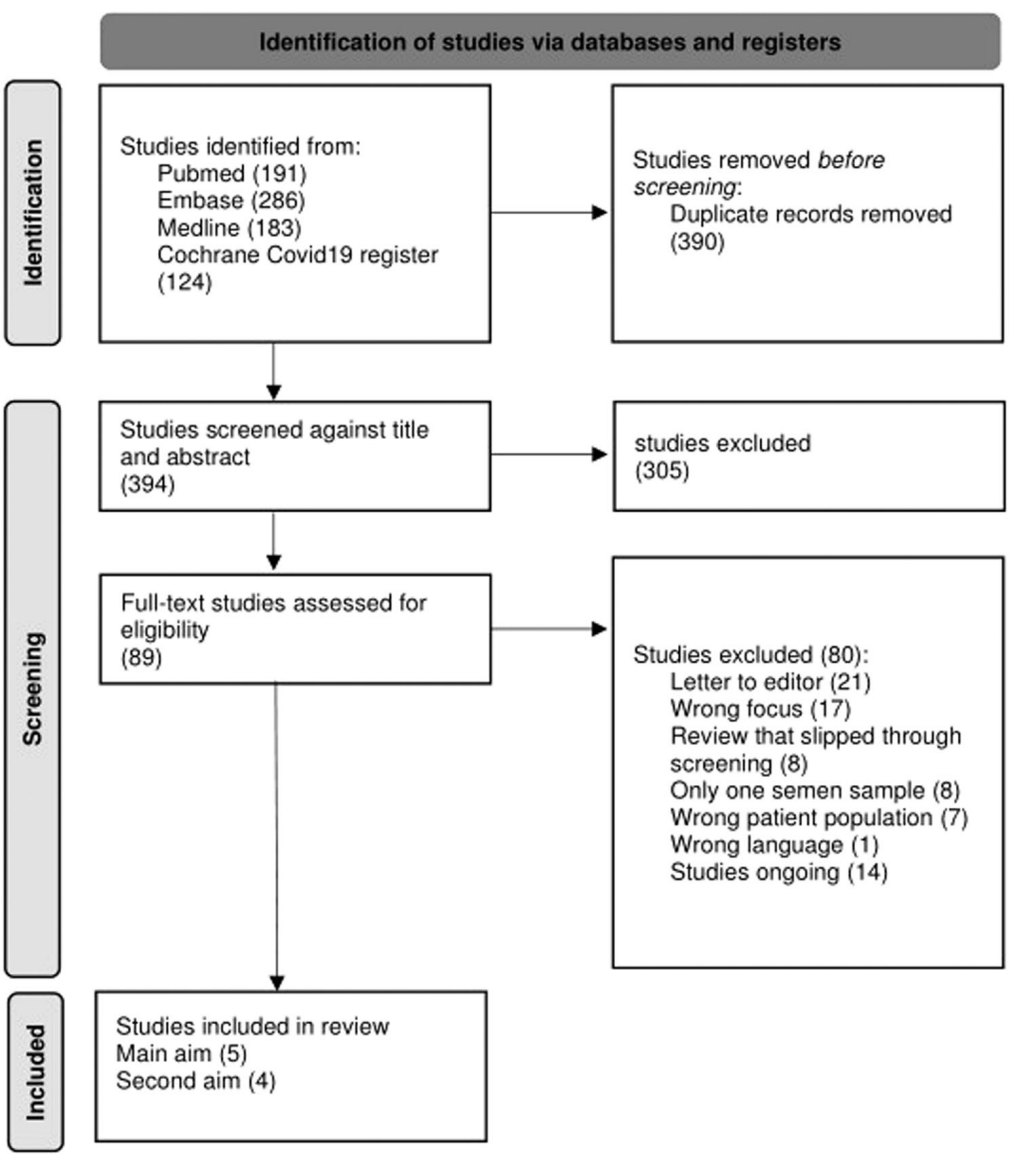
Preferred reporting items for systematic reviews and meta‐analysis flowchart illustrating the process of selecting the articles.

As illustrated in Figure 1, half of the studies identified were removed as duplicates, leaving 394 articles for the title and abstract screening. Three hundred and five (305) articles were then excluded, including other review articles. The reasons for the exclusion of each article during full‐text reading can be seen in Figure 1. Twenty one of the studies identified were not original studies but comments on other studies and were therefore excluded. Fourteen studies were not finished but simply registered on online databases such as Clinicaltrials.gov and did not yet have any articles in the press on the study, so they could not be included in the review.

Nine studies satisfied the aims and criteria and were therefore included in the final review.

### 2.5 Data extraction

The main semen parameters analyzed by most of the articles were used to create Tables 1 and 2.

**Table 1:** Semen quality in the five studies included data before as well as after COVID‐19 infection

Article		Population size	Days after the positive test (mean)	Volume (ml)		*p*‐Value	Sperm concentration (million/ml)		*p*‐Value	Progressive motility (%)		*p*‐Value	Total motility (%)		*p*‐Value
				Before	After		Before	After		Before	After		Before	After	
Ma et al.^9^		3	78.5 (56–109)^a^	4.33 ± 1.328	4.63 ± 1.115	0.668	49.27 ± 30.42	53.1 ± 45.61	0.7086	30.47 ± 12.77	26.63 ± 11.27	0.245	43.33 ± 19.72	30.4 ± 13.06	0.2822
Gul et al.^10^		29	137.6 ± 40.8^b^	2.23 ± 1.11	2.58 ± 1.01	0.138	39.67 ± 40.45	47.52 ± 60.84	0.573	26.62 ± 12.59	29.24 ± 15.49	0.107	31.23 ± 15.06	33.97 ± 19.07	0.334
Koç and Keseroglu^11^		21	51 (37–89)^a^	**3 (1–8)** a	**2.5 (1.5–5)** a	0.005	42 (2–148)^a^	35.9 (4–126.5)^a^	0.689	**35.1** ± 21.7	**21.8** ± **15.9**	**<0.001**	**48.6** ± **22.1**	**34.7** ± **20.7**	**0.001**
Pazir et al.^12^		24	111.5^a^	3.6 ± 1.6	3.5 ± 1.5	0.56	42.6 ± 18.0	35.3 ± 20.2	0.06	34.5 ± 1.5	28.9 ± 9.1	0.14	**45.8** ± **5.0**	**40.4** ± **10.9**	**0.01**
Erbay et al.^13^	Mild symptoms	26	119.42 (94–144)^b^	3.24 ± 1.6	3.08 ± 0.8	0.548	32.24 ± 12.8	28.62 ± 12.4	0.055	**28.81** ± **9.7**	**20.92** ± **9.1**	**0.002**	**48.69** ± **12.1**	**33.41** ± **12.3**	**0.002**
	Moderate symptoms	43	127.66 (96–190)^b^	**3.34** ± **1.1**	**2.74** ± **0.9**	**<0.001**	**35.01** ± **14.1**	**30.06** ± **17.2**	**0.008**	**30.16** ± **12.1**	**21.4** ± **10.1**	**<0.001**	**50.74** ± **13.4**	**31.42** ± **13.3**	**<0.001**

*Note*: Studies with data before and after covid‐19, before = before covid19, and after = after covid19.

Results that are in bold are statistically significant (*p* < 0.05). Data are presented as mean value ± standard deviation unless specified otherwise.

^a^ Result presented as median with interquartile range.

^b^ Median and interquartile range from clinical recovery and not the positive test.

**Table 2:** Semen quality in the three studies included two sets of data after the COVID‐19 infection

Article	Population size	Days between the two semen samples	Volume (ml)		*p*‐Value	Sperm concentration (million/ml)		*p*‐Value	Progressive motility (%)		*p*‐Value	Total motility (%)		*p*‐value
			First sample	Second sample		First sample	Second sample		First sample	Second sample		First sample	Second sample	
Best et al.^14^	5	91	2.18 ± 0.249	1.3 ± 0.6819	0.0958	11.63 ± 9.398	18.6 ± 3.715	0.2524				2.8 ± 6.261	21.6 ± 31.42	0.2348
Falahieh et al.^15^	20	106	3.8 ± 1.2	4.1 ± 1.3	0.4389	47.6 ± 21.9	52.1 ± 24.3	0.5422	**30.6** ± **8.2**	**44.1** ± **9.9**	**<0.0001**	**32.8** ± **8.9**	**47.5** ± **9.8**	**<0.0001**
Guo et al.^16^	22	31	3 (2.3–4.0)^a^	3 (3.0–5.0)^a^	0.2813	**39.2 (26.6)** a	**59 (45.7**–**112.5)** a	0.0066	35.1 (20.4–42.0)^a^	37 (29.4–50.1)^a^	0.5661	39.8 (27.0–50.5)^a^	42.2 (34.7–54.1)^a^	0.6237

*Note*: Maleki et al.^17^ presented no data and were not included in the table.

Studies with two semen samples after the positive covid test.

Results that are in bold are statistically significant (*p* < 0.05), and data are presented as mean value ± standard deviation unless specified otherwise.

^a^ Result presented as median with an interquartile range.

Where the articles used the same measures, it was possible to compare the results directly.

If an article presented no statistical data, an effort was made to find the original individual data in the article or as supplementary data in the study. We used GraphPad Prism 9.3.1 to perform statistical analysis on these data.

Paired Student's *t*‐test was used to compare results within groups. Data are presented as means ± standard deviations. QQ plot was used to check for normality.

Where original data were not found, the results were not included in the tables, but their conclusions were still included in the comparison.

When additional results were found relevant, they were included and commented on.

## 3 RESULTS

### 3.1 Included articles

**Figure 2:**
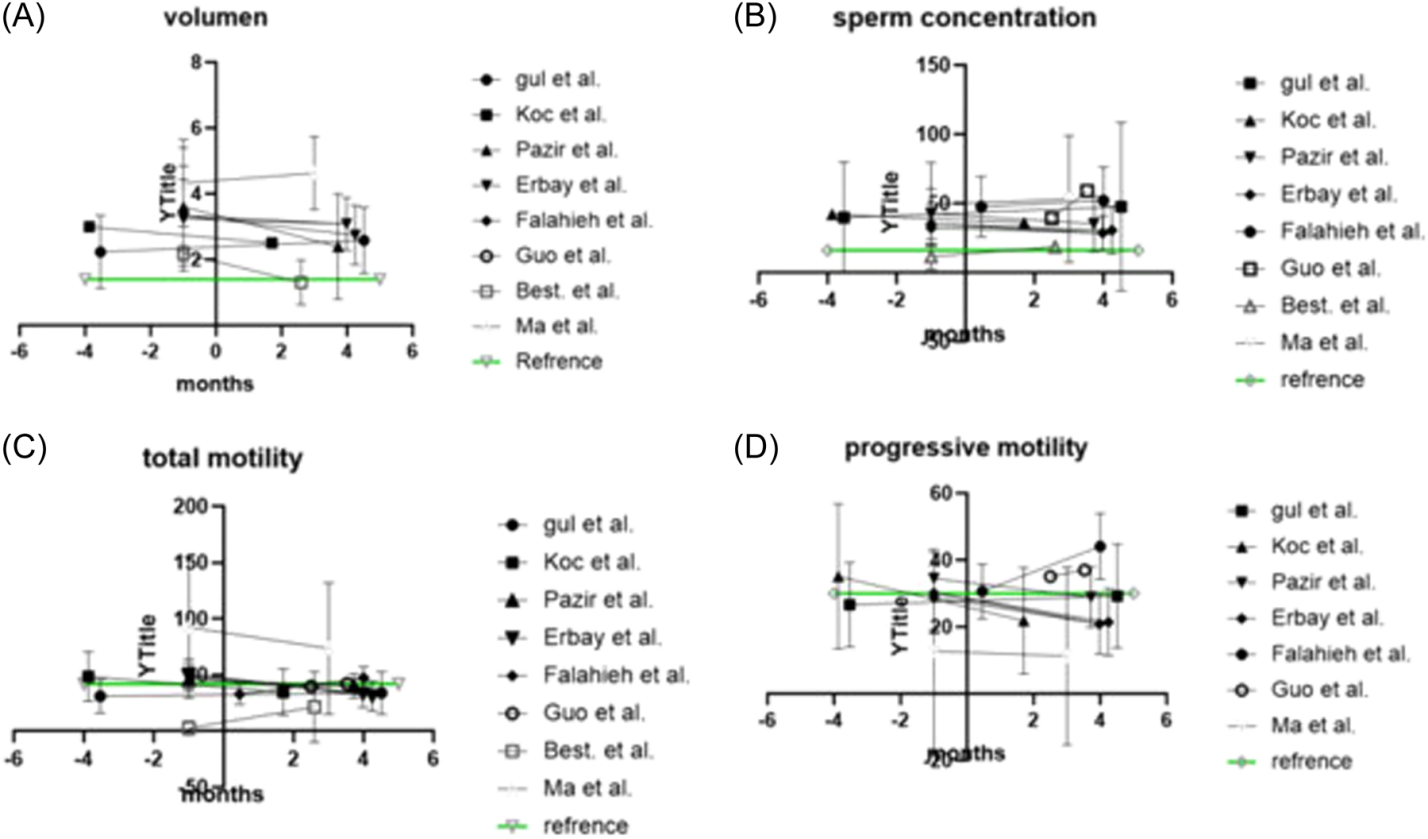
Results from Tables 1 and 2 showed together for each category, which illustrates the changes in the semen parameters over time in the eight articles with data. (A) Change in volume (ml), (B) change in sperm concentration (million/ml), (C) change in total motility (%), and (D) change in progressive motility (%). Reference is the references according to World Health Organization.^2^

Five articles had pre‐ and post‐COVID‐19 infection data and four articles had more than one set of post‐COVID‐19 infection data but no pre‐COVID‐19 data. The two groups of articles were used to create Table 1 and Table 2, respectively. Parameters in Tables 1 and 2 include semen volume, sperm concentration, total motility, and progressive motility. These parameters are illustrated in Figure 2A–D, which uses data from Tables 1 and 2.

One article in each group only had individual patient data available in the article^9^ or as supplementary data,^14^ and analytical data could then be determined and included.

It was not possible to find the original data on patients in the last article^17^ in the group with two semen samples collected after COVID‐19 infection. Because of the different focus, the results were only presented as a figure, making it difficult to compare the outcomes directly in Table 2.

### 3.2 The five articles with pre‐ and post‐COVID‐19 data

In four of the five articles with pre‐ and post‐COVID‐19 data the patients were either found via the database of a fertility clinic or via data available otherwise from being examined previously for their fertility status.^10^, ^11^, ^12^, ^13^ The last article by Ma et al.^9^ focused on a single sample from each COVID‐19 patient compared to a control group, and, in addition, three patients had data from previous semen analyses.^9^ Two of these three patients presented with two semen analyses before SARS‐CoV‐2 infection but with no exact date of sample collection. With the knowledge that events and lifestyle changes could affect semen quality,^18^, ^19^, ^20^, ^21^ it was assumed that the most accurate to compare with, was the most recent one.^9^

In three articles,^10^, ^11^, ^12^ the number of participants was between 21 and 29, while in the article by Erbay et al.,^13^ there were 69 patients, divided into 26 with mild symptoms and 43 with moderate symptoms.

In these four articles,^10^, ^11^, ^12^, ^13^ the criteria for exclusion included any known event that might lead to reduced fertility, like previous urogenital infection, testicular diseases, azoospermia, or oligozoospermia. One article mentioned hormonal drug use as an exclusion criterion,^13^ and one described no exclusion criteria at all.^9^ Lastly, one article excluded patients who had been sick with a non‐COVID‐19 febrile illness in the last 3 months.^12^

When looking at the duration of time between pre‐ and post‐COVID‐19 semen sample analyses, two articles mentioned that the data were obtained within a year or 2,^10^, ^13^ while the rest were unclear. Two articles described the authors to have waited at least 3 months after confirmation of patients’ COVID‐19 infection to collect their semen samples.^10^, ^12^ A third article waited 3 months after the patients were proven to have recovered, according to their nasopharyngeal swab sample tests, before semen sampling.^13^

Gul et al.^10^ found no statistical difference between the semen parameters before and after COVID‐19 infection, and the result did not change when the hospitalization time and medication were considered.^10^

Pazir et al.^12^ adopted almost the same time interval until testing as Gul et al.,^10^ and there was a significant decrease in total motility (*p* = 0.01) and total motile sperm count (*p* = 0.02).^12^

In addition, Pazir et al.^12^ divided the patients into groups with and without fever as a symptom, to see if the negative effect on semen parameters could be explained by febrile episodes. The group with fever showed no significant difference between their pre‐ and post‐COVID‐19 semen parameters, whereas only total motility decreased significantly after COVID‐19 in the group without fever.^12^ Koç and Keseroglu^11^ waited 37–89 days (median 51) between a positive COVID‐19 test and semen analysis, with the semen volume, progressive motility, and total motility after SARS‐CoV‐2 infection significantly decreasing (*p* < 0.05) compared to the same semen parameters before SARS‐CoV‐2 infection. Furthermore, the normal sperm morphology significantly decreased after COVID‐19 infection, whereas the percentage of immotile sperm significantly increased.^11^ Comparing the results to the WHO^8^ reference in Figure 2, we see that both the progressive and total motility were above normal before COVID‐19 infection but fell below the “cut‐off” after the infection.^11^

Erbay et al.^13^ stood out by dividing the patients into groups according to their symptoms. In the group with mild symptoms, progressive and total motility decreased significantly (*p* = 0.002 in both), while in the group with moderate symptoms, all parameters decreased significantly (*p* < 0.05).^13^

In addition to these results, which are illustrated in Table 1, the group with mild symptoms showed a significant decrease in vitality (*p* = 0.03), while the moderate symptom group showed a decrease in both the total sperm number and vitality (*p* = 0.001 for both).^13^ The article by Ma et al.^9^ was the one with only three patients, and the authors found no significance in the changes in semen parameters before and after the COVID‐19 tests.

### 3.3 The four articles with two sets of post‐COVID‐19 test data

In three^15^, ^16^, ^17^ of the four articles with post‐COVID‐19 test data, the patients were recruited due to hospitalization at some point because of COVID‐19. In the last article, the patients were identified using an electronic medical record search for men who had tested positive for SARS‐CoV‐2 infection.^14^ The number of participants varied greatly in this group of articles, ranging from only five patients in Best et al.^14^ to 84 patients in Maleki and Tartibian^17^ The other two articles had 20 and 22 patients.^15^, ^16^

All four articles excluded patients with known infertility or other factors that are known to increase the risk of infertility, like earlier cryptorchidism or scrotal surgery, abnormal secondary sexual characteristics and small testicular size, or a history of mumps or sexually transmitted infections. Two articles only included patients with proven fertility, like men whose wives had given birth to healthy children within the previous two years.^15^, ^17^ Two articles included COVID‐19 negative control groups.^16^, ^17^

The time between diagnosis and first and second semen sample collection differed in all of the articles.

The article by Falahieh et al.^15^ had 20 patients, and semen samples were analyzed in the active stage of SARS‐CoV‐2 infection (Day 14), and then again after 120 days. The authors found that the progressive motility and total sperm motility of the first sample after COVID‐19 diagnosis were below normal, but both parameters increased significantly (*p* < 0.0001 for both) on Day 120 and reached the normal range (see Figure 2).^15^ Furthermore, the sperm morphology improved significantly (*p* = 0.0002) on Day 120 but did not reach the normal value according to the WHO criteria.^8^

Guo et al.^16^ waited for a median of 76 (interquartile range [IQR]: 73–86.5) days from the onset of symptoms until the first semen sample collection, which was also 56 (IQR: 49–72) days from discharge from the hospital. The second sample was collected 29 (IQR: 28–32.8) days after the first one. The authors found a statistically significant increase in sperm concentration (*p* = 0.0066) between the two samples. In addition to the characteristics enumerated in Table 2, the total sperm count and motile sperm count increased significantly (*p* = 0.0029 and *p* = 0.0391, respectively).^16^

The article by Maleki and Tartibian^17^ had the largest patient population: 84 in the COVID‐19 group. The patients were recruited 24 h after discharge from the hospital, and semen samples were collected that day, with sample collection continuing with 10‐day intervals until Day 60. The mean time between COVID‐19 diagnosis and first semen sample collection was 13.2 ± 4.9 days.^17^ No significant changes were observed in semen volume, sperm concentration, or sperm morphology during the follow‐up periods.^17^ Further, this article included a control group, and, compared with this group, the COVID‐19 group had significantly lower semen volume, sperm concentration, number of spermatozoa with progressive motility, and sperm morphology in all follow‐up samples.^17^

The last article in this group, by Best et al.,^14^ compared semen samples from COVID‐19 patients with those from a control group. Out of 30 COVID‐19 patients that delivered first semen samples, only five patients delivered follow‐up samples. The median time between diagnosis and first semen sample collection was 37 days, with an IQR of 23 days, and the median duration of time from first sample collection to second sample collection was 91 days (IQR: 61).^14^ No significant change was found when comparing the first semen sample with the follow‐up samples.^14^

## 4 DISCUSSION

Our findings show a correlation between SARS‐CoV‐2 infection and a decrease in semen parameters for a limited time after testing positive for COVID‐19.

One of the earliest preoccupations of researchers was demonstrating if COVID‐19 could be found in the male genital tract to establish if the virus could be contracted by this route. In a review by Omolaoye et al.^22^ it was shown that most studies did not find the SARS‐CoV‐2 RNA in the semen, although a few did, and no specific connection was found when comparing different degrees of symptoms in different study results. These findings are supported by a review by Segar et al.,^4^ who suggested that because the SARS‐CoV‐2 viral RNA was not found in the testicular tissues, the effect on fertility must be linked to immune responses which often include fever.^4^

The results from Pazir et al.,^12^ meanwhile, contradicted this hypothesis about fever. Pazir et al. found no statistically significant difference between pre‐and post‐COVID‐19 semen parameters in the group with fever, whereas they found a significant decrease in total motility in the group without fever (*p* = 0.03).^12^

The article did not describe the reference value used as a cut‐off for temperature, the temperature range within the group with fever, and the lengths of febrile episodes.^12^

These results could be explained by the possibility of coincidence in them, because there were only 12 patients in each group or because the fever was not high enough to lead to the affection of germ cells and inhibition of spermatogenesis.^23^ The study by Maleki and Tartibian^17^ found that 98.8% of its COVID‐19 population had a fever as a symptom. The results of COVID‐19 patients, compared with a control group, showed significantly lower values for all semen parameters at all times from Day 1 to Day 60. The authors suggested that the high fever caused by COVID‐19 might be responsible for this result.^17^

In the study by Koç and Keseroglu,^11^ in which only 9.5% of the patients presented with fever, a statistically significant decrease was found in semen volume (*p* = 0.005), progressive motility (*p* < 0.001), and total motility (*p* = 0.001) in the samples collected after positive COVID‐19 diagnosis, compared to those collected before. In the study by Erbay et al.,^13^ 69.3% and 72.1% of patients had a fever in the groups with mild symptoms and moderate symptoms respectively, and significant decreases in all parameters were found in the group with moderate symptoms whereas there were significant decreases only in progressive and total motility in the mild group.^13^ The article by Guo et al.^16^ had 85% of patients who presented with fever symptoms; the study compared the semen parameters of COVID‐19 patients with those of a control group and found significantly lower values in the COVID‐19 group in sperm concentration (*p* = 0.0115), progressive motility (*p* = 0.0233) and total motility (*p* = 0.028). The authors thus concluded that fever might be the reason for the poor values of the parameters.^16^

Comparing the results of the above studies with those of the studies by Maleki and Tartibian^17^ and Pazir et al.,^12^ it appears that fever reduces semen quality, but other factors play a part too.

Another point where the articles differed from each other concerns the amount of time between COVID‐19 diagnosis and subsequent semen sample collection. For the articles with pre‐ and post‐COVID‐19 data, Koç and Keseroglu^11^ waited 37–89 days (median 51) from diagnosis until semen sampling, and they found significant decreases in the semen parameters. In the two studies in which ejaculates were delivered at least 3 months after COVID‐19 diagnosis,^10^, ^12^ no significant changes were found in semen parameters before and after SARS‐CoV‐2 infection, except for the decrease in total motility in Pazir et al.^12^ (*p* = 0.01). The difference in the results from Pazir et al.^12^ and Gul et al.^10^ could be explained by the fact that Pazir et al.^12^ did not take the duration of illness and symptoms into account, whereas Gul et al.^10^ considered the months after clinical recovery. The last article in this group^13^ also took the clinical recovery period into account and waited around 3–4 months from then until sample collection; the authors found significant decreases in all parameters in the group with moderate symptoms and significant decreases only in progressive motility and total motility in the mild group. These results suggest that for some time after SARS‐CoV‐2 infection spermatogenesis might be impaired but will become normal again. Judging by the results of Pazir et al.^12^ and Erbay et al.,^13^ some parameters seem to take longer to recover after being affected by COVID‐19, e.g. the number of motile sperm cells.

The group of articles with more than one semen sample collected after a positive COVID‐19 diagnosis seems to support this conclusion. Maleki and Tartibian^17^ took less than two months after the patient's discharge from the hospital before collection of the first sample after COVID‐19 diagnosis, and they found no significant differences in the pre‐and post‐COVID‐19 semen parameters in the COVID‐19 group, but, compared with the control group, the values were significantly lower in the COVID‐19 group.^17^ Falahieh et al.^15^ and Gou et al.^16^ collected the first samples less than three months after diagnosis and the second samples more than three months after diagnosis. There was an increase in each parameter from the first sample to the second in both studies and statistically significant increases in progressive and total motility in Falahieh et al.^15^ and in sperm concentration in Guo et al.^16^

Besides showing that SARS‐CoV‐2 infection affects spermatogenesis, these data tend to suggest that the damage is reversible. After more or less three months, which is the average duration for spermatogenesis, the semen parameters return to normal.

This review has both strengths and limitations. The populations in the included studies are quite comparable: all patients fell within the age range of 20–50 years and had BMI between 23 and 27 kg/m^2^. Further, most of the studies exclude and stratify for possible confounders as enumerated in the method section.

The time perspectives in the articles vary, which makes them hard to compare directly, but this also means that they cover a wider time span. By comparing them, this review has been able to suggest how semen parameters vary during and after SARS‐CoV‐2 infection.

The severity of patient symptoms also varied in different studies, with some patients expressing mild symptoms and others needing to be hospitalized. Medications varied from no medication to multiple different medications. The severity of the illness had no impact on the variations in the results when comparing the articles. Regarding to medication, two articles mentioned that patients were treated with corticosteroids,^16^, ^17^ which might have affected the results. Corticosteroids are known to have a negative impact on semen quality,^24^ so in the study by Maleki and Tartibian^17^ where it was used by 44% of patients, it could have affected the results, but as other articles did not treat patients with this drug, it does not have an impact on the conclusion given in this review.

Smoking could affect semen quality^18^ and should be taken into account as a likely confounder. Only one study considered smoking as an exclusion criterion,^15^ but it still found a significant increase in semen parameters when comparing samples collected during the acute phase of the illness to samples collected over three months after the infection. Some articles did not mention the smoking status of the patients, while some mentioned it (ranging from 6.9% to 61.5%) but did not comment further on it. It would have been interesting if the studies that included smokers divided the patients into smokers and nonsmokers to evaluate any differences in the severity of the illness or in the results of semen analysis between the two groups.

This review, although not a systematic review, applied the search string to four different databases in an effort to include all relevant material in the literature.

From the nine articles, it may be suggested that spermatogenesis might be affected for at least the time it takes to recover from COVID‐19, and then produce new spermatozoa. Why male fertility is affected after SARS‐CoV‐2 infection is still unclear, with several components probably influencing this. Because only a few studies are available on this subject, with two including less than five participants, there is a need for more research. New research should include larger sample sizes and follow patients over a longer time, to prove or disprove the conclusions in this review.

It may be relevant to also analyze the likely confounders mentioned.

As COVID‐19 continues to affect populations worldwide, it is important that fertility clinics consider the possible effects of the virus on semen quality when evaluating a man with low semen parameters before deciding what help he needs or whether to request a new semen sample after some months.

## AUTHOR CONTRIBUTIONS

Jens Fedder, Kathrine Tufvesson, and Laura Catalini conceptualized the study. Kathrine Tufvesson performed the search, and Laura Catalini and Jens Fedder verified the search results. Kathrine Tufvesson and Laura Catalini extracted the data and developed the tables and figures. The original draft was made by Kathrine Tufvesson and was subsequently edited by all authors. All authors have read and approved the final version of the manuscript, had full access to all of the data in this study, and taken complete responsibility for the integrity of the data and the accuracy of the data analysis.

## CONFLICT OF INTEREST

The authors declare no conflict of interest.

## TRANSPARENCY STATEMENT

Jens Fedder affirms that this manuscript is an honest, accurate, and transparent account of the study being reported; that no important aspects of the study have been omitted; and that any discrepancies from the study as planned (and, if relevant, registered) have been explained.

## Data Availability

All the main data and materials are available in the article. Additional data are available upon request.
